# “Lactose free” depression- Antidepressant with and without lactose registered in Croatia

**DOI:** 10.1192/j.eurpsy.2023.1755

**Published:** 2023-07-19

**Authors:** D. Svetinović, I. Barun, S. Vuk Pisk, I. Filipčić, V. Grošić

**Affiliations:** Department of Integrative psychiatry, University psychiatric hospital Sveti Ivan, Zagreb, Croatia

## Abstract

**Introduction:**

Depression is a common illness worldwide, with an estimated 3.8% of the population affected, including 5.0% among adults and 5.7% among adults older than 60 years. Lactose intolerance affects 70% of the world population. With both conditions being common there are a lot of people having both lactose intolerance and depression. People with lactose intolerance are unable to fully digest lactose. As a result, they have diarrhea, bloating and gas after eating or drinking dairy products. Lactose is one of the most used excipients in drug formulations and is ofteny overlooked when prescribed.

**Objectives:**

To quantify and identify the amount of lactose in medications used for the treatment of depression and to identify ‘lactose-free’ medication registered in Croatia.

**Methods:**

Medications used for the treatment of depression were identified from the Agency for medicinal products and medical products of Croatia (HALMED). Their formulation including excipients was obtained from the Agency.

**Results:**

Wide range of antidepressants contains lactose. We have quantified the lactose amount using information on medicinal products with marketing authorisation granted by HALMED.

**Table:** 

Antidepressant	Brand name and dosage	Lactose per tablet (mg)
Sertraline	Sonalia ® 50 mg	19.80mg
Paroxetine	Paroksetin PharmaS®	10mg
Escitalopram	Escital ® 10mg	117.8mg
	Elicea ® 5mg	51.3mg
Citalopram	Citalon ® 20mg	23mg
Mirtazapine	Calixta ® 15mg	44.4mg
	Mirzaten ® 30mg	120.56mg
	Mirzaten Q tab ® 15mg	35.63mg

**Conclusions:**

With this research, we have pointed out a high proportion of the most commonly prescribed antidepressants that contain lactose. Considering the high proportion of the general population with lactose intolerance, we have pointed out the importance of knowing the data that antidepressants do not contain lactose in order to choose an adequate therapy for our patients, while not causing them discomfort that will further reduce the effectiveness of the therapy, as well as increase the percentage of those who due to the side effects of the drug, they stop taking the therapy. This research will help clinicians in their daily work to choose the most optimal therapy for their patients. With this study, we will give doctors a list of medications for depression treatment without lactose. With this study, we will give doctors a list of medications for depression treatment without lactose.

**Disclosure of Interest:**

None Declared

